# Blood DNA methylation and liver cancer in American Indians: evidence from the Strong Heart Study

**DOI:** 10.1007/s10552-023-01822-8

**Published:** 2023-11-27

**Authors:** Monique Slowly, Arce Domingo-Relloso, Regina M. Santella, Karin Haack, Daniele M. Fallin, Mary Beth Terry, Dorothy A. Rhoades, Miguel Herreros-Martinez, Esther Garcia-Esquinas, Shelley A. Cole, Maria Tellez-Plaza, Ana Navas-Acien, Hui-Chen Wu

**Affiliations:** 1 Department of Environmental Health Sciences, Columbia University Mailman School of Public Health, New York, USA; 2 Department of Chronic Diseases Epidemiology, National Center for Epidemiology, Carlos III Health Institute, Madrid, Spain; 3 Department of Statistics and Operations Research, University of Valencia, Valencia, Spain; 4 Herbert Irving Comprehensive Cancer Center, Columbia University Medical Center, New York, NY USA; 5 Population Health Program, Texas Biomedical Research Institute, San Antonio, TX USA; 6 Department of Mental Health, Johns Hopkins University, Baltimore, MD USA; 7 Department of Epidemiology, Johns Hopkins University, Baltimore, MD USA; 8 Department of Epidemiology, Columbia University Mailman School of Public Health, New York, NY USA; 9 Department of Medicine, Stephenson Cancer Center, University of Oklahoma Health Sciences, Oklahoma City, OK USA; 10 Bioinformatics Unit, Institute for Biomedical Research INCLIVA, Valencia, Spain; 11 Universidad Autónoma de Madrid, Madrid, Spain; 12 CIBERESP (CIBER of Epidemiology and Public Health), Madrid, Spain

**Keywords:** American Indian, Liver cancer, Circulating leukocyte subtypes, CpG site, DNA methylation, Strong Heart Study

## Abstract

**Purpose:**

Liver cancer incidence among American Indians/Alaska Natives has risen over the past 20 years. Peripheral blood DNA methylation may be associated with liver cancer and could be used as a biomarker for cancer risk. We evaluated the association of blood DNA methylation with risk of liver cancer.

**Methods:**

We conducted a prospective cohort study in 2324 American Indians, between age 45 and 75 years, from Arizona, Oklahoma, North Dakota and South Dakota who participated in the Strong Heart Study between 1989 and 1991. Liver cancer deaths (*n* = 21) were ascertained using death certificates obtained through 2017. The mean follow-up duration (SD) for non-cases was 25.1 (5.6) years and for cases, 11.0 (8.8) years. DNA methylation was assessed from blood samples collected at baseline using MethylationEPIC BeadChip 850 K arrays. We used Cox regression models adjusted for age, sex, center, body mass index, low-density lipoprotein cholesterol, smoking, alcohol consumption, and immune cell proportions to examine the associations.

**Results:**

We identified 9 CpG sites associated with liver cancer. cg16057201 annotated to *MRFAP1*) was hypermethylated among cases vs. non-cases (hazard ratio (HR) for one standard deviation increase in methylation was 1.25 (95% CI 1.14, 1.37). The other eight CpGs were hypomethylated and the corresponding HRs (95% CI) ranged from 0.58 (0.44, 0.75) for cg04967787 (annotated to *PPRC1*) to 0.77 (0.67, 0.88) for cg08550308. We also assessed 7 differentially methylated CpG sites associated with liver cancer in previous studies. The adjusted HR for cg15079934 (annotated to *LPS1*) was 1.93 (95% CI 1.10, 3.39).

**Conclusions:**

Blood DNA methylation may be associated with liver cancer mortality and may be altered during the development of liver cancer.

**Supplementary Information:**

The online version contains supplementary material available at 10.1007/s10552-023-01822-8.

## Introduction

The incidence of primary liver cancer in the US has steadily increased by > 2% annually, even as most other cancers are declining [1]. Liver cancer incidence varies by racial/ethnic groups, with the highest incidence rate (per 100,000) in American Indians/Alaska Natives (AI/AN) (15.7), followed by Hispanics (13.5), Asian/Pacific Islanders (12.6), Blacks (11.0), and Whites (7.1) between 2013 and 2017 [1]. The major risk factors of liver cancer include metabolic factors, hepatitis virus infection, excess alcohol use, smoking, and genetic factors; however, those factors only account for ~ 60% of liver cancer [2]. In addition to environmental factors, individual susceptibility is also associated with liver cancer [3–6]. Identifying healthy individuals with a high-risk of disease may facilitate the establishment of interventions to reduce cancer incidence and mortality.

DNA methylation can regulate gene expression by affecting how DNA interacts with both chromatin proteins and specific transcription factors [7]. Population epigenetic studies have focused on identifying differences in DNA methylation between specific exposures, or diseases in accessible tissues such as peripheral blood [8–12]. These studies showed that epigenetics is an important aspect of human health and disease due to variability in epigenetic modification and its potential for mediating the interaction between environmental exposures and phenotypic outcomes [9, 13–15]. Limited epigenome-wide association studies (EWAS) using Infinium HumanMethylation450k arrays identified selected DNA methylation marks related to liver cancer, using a nested case–control study design [16, 17]. However, these liver cancer-related markers have not been assessed in AI/AN populations.

The development of liver cancer is associated with prolonged inflammation caused by viral infections, toxins, or fatty liver disease [18]. Chronic inflammation can generate an immunosuppressive microenvironment, and cause tumor antigen tolerance, leading to carcinogenesis [19]. Understanding immune cell profiles preceding cancer can provide potential prevention and early diagnosis strategies.

The goals of this study are to conduct an epigenome-wide association study (EWAS) to identify differentially methylated positions (DMPs) associated with liver cancer and to validate the DMPs associated with liver cancer from previous studies [16, 17]. In addition, we determined associations between circulating leukocyte subtypes and risk of liver cancer. We used prospectively collected peripheral blood samples from the Strong Heart Study, a large cohort of American Indian adults from the Southwest, Northern Plains and Southern Plains [20]. We measured DNA methylation using MethylationEPIC BeadChips and estimated the proportions of 6 leukocyte subtypes (B cells, natural killer cells, monocytes, CD8+ and CD4+T cells, and granulocytes) using a computational approach developed by Houseman [21].

## Materials and methods

### Study population and design

The Strong Heart Study (SHS) enrolled a cohort of 4,549 men and women aged 45–75 years old who were members of 13 tribes from Arizona, Oklahoma, and North Dakota and South Dakota between 1989 and 1991 [20]. Participants were interviewed by trained and certified nurses and medical examiners to collect information on sociodemographic factors, medical history and smoking status (never, former, current) using a structured questionnaire. Participants having smoked ≥ 100 cigarettes in their lifetime and smoking at the time of the interview were classified as current smokers. Participants who smoked ≥ 100 cigarettes in their lifetime but who were not smoking at the time of interview were classified as former smokers. Participants who have never smoked or who have smoked < 100 cigarettes in their lifetime were classified as never smokers. Current alcohol consumption was defined as any alcohol use within the past year. Former alcohol consumption was defined as no use of any alcohol during the past year but previous use of > 12 drinks of alcohol. No alcohol consumption was defined as < 12 drinks in the participant’s lifetime. A threshold of 100 cigarettes to determine smoking status and 12 drinks to determine alcohol consumption status were used according to the definitions provided by the National Health Interview Survey. The nurses and medical examiners conducted a physical exam including anthropometric measures (height and weight to measure body mass index (BMI)) and the collection of fasting blood and spot urine samples. Buffy coats from fasting blood samples were stored at −70 °C. DNA from white blood cells was extracted and stored at MedStar Health Research Institute.

For this prospective study of liver cancer mortality, we used blood DNA methylation data generated for a prior study whose main focus was hematopoietic, solid and overall cancers [22]. The criteria used for selection of the study participants has been reported previously and included participants free of cardiovascular disease at baseline, not missing data on risk factors for cardiovascular disease, and with sufficient DNA available for the analysis of blood DNA methylation [22, 23]. One tribe declined participation. The sample size for this study was 2,324.

### Ascertainment of liver cancer death

The SHS uses tribal records, death certificates, medical records, and direct annual contact with participants and their families to assess health outcomes and vital status over time. Liver cancer mortality was assessed by death certificates and/or medical chart reviews. International Classification of Diseases, 9th Revision (ICD-9), code (ICD-9 code: 155) was used to define outcomes. Using death certificates and medical records obtained through 2017, we identified a total of 21 cases of liver cancer.

### Microarray DNA methylation determinations

Blood DNA was bisulfite-converted with the EZ DNA methylation kit (Zymo Research) according to the manufacturer’s instructions. Bisulfite converted DNA was measured using the Illumina MethylationEPIC BeadChip (850 K) at Texas Biomedical Research Institute. Samples were randomized across and within plates to remove potential batch artifacts and confounding effects. Replicate and across-plate control samples were included on every plate. Data were read in six different batches (of ~ 400 individuals each) and combined using the R package minfi. The methylation data processing procedures have been described elsewhere [22]. In brief, methylation measures with a detection *p*-value > 0.01 indicated unreliable measurement. Individuals with low detection *p*-values, cross-hybridizing probes, probes located in sex chromosomes and SNPs (Single Nucleotide Polymorphisms) with minor allele frequency > 0.05 were excluded. Single sample noob normalization and regression on correlated probes normalization were conducted following Illumina’s recommendations for preprocessing. The final number of CpG sites available for analysis was 788,368.

We used the Houseman method to estimate composition of the 6 leukocytes subtypes (B cells, natural killer cells, and CD8+ and CD4+T cells (lymphoid lineage), and monocytes and granulocytes (myeloid lineage)) for each sample [21].

### Statistical methods

We conducted an EWAS analysis using linear models as implemented by the R package limma [24] to evaluate the individual association of each CpG site (Beta value) with liver cancer mortality, without accounting for time-to-event and identify DMPs by liver cancer case vs. non case status. Empirical Bayes shrinkage [25] was used to shrink the standard errors towards a pooled estimate to achieve more robust inference. *p*-value was adjusted for multiple comparisons using Benjamini and Hochberg, or the false discovery rate (FDR) approach [26]. We used 0.07 as the significance cut-off for FDR p-values in the EWAS analysis due to the small number of cancer cases and limited power for the high-dimensional model.

#### Candidate CpG marker analysis

Participant characteristics were stratified by liver cancer status. We used the χ^2^ test for categorical variables and ANOVA for continuous variables to assess the difference of selected characteristics between liver cancer cases and non-cases. ANOVA was also used to determine the difference in immune cell proportion between cases and non-cases. We calculated follow-up from the date of baseline examination to the date of death or 31 December 2017, whichever occurred first. We ran Cox proportional hazards models using follow-up time as timescale to determine the hazard ratios (HRs) for each DMP identified as significant from the EWAS as well as from previous, nested case–control studies [16, 17]. Our HRs and confidence intervals are standardized by one standard deviation (SD) increase in methylation (Beta value) to control for small sample size and outliers. We classified CpG sites as hypermethylated or hypomethylated dependent on the average beta values on the respective CpG site in cases compared to non-cases. Analyses were conducted in R (version 4.2.1), using the package “survival”. We conducted a sensitivity analysis, adjusting for pack-years of smoking for a more granular measurement of smoking behavior. We found that the direction and effect size of the hazard ratios in these models corresponded with our models using a categorical smoking variable. However, continuous measurements of smoking behavior were not recorded for the entire study population (*n* = 2,235). As heavy drinking of alcohol was recorded for a small subsect of the study population (20%), we were unable to run this model.

We analyzed candidate CpGs from two studies examining the association between HCC and DNA methylation. Kao et al. identified three methylation signatures, cg00300879, cg06872964, and 07080864, in peripheral leukocytes that could track the disease progression of HCC in Hepatitis B virus (HBV) positive participants in Taiwan [16]. Lubecka et al. identified 9 probes that distinguished HCC cases from cirrhotic controls in HBV negative participants. We assessed the CpG sites associated with 19 probes initially assessed in the Lubecka et al. study [17]. Both studies used Illumina HumanMethylation 450 K Beadchip arrays to assess DNA methylation in leukocytes. The 850 K array used in this study was able to provide methylation data for 7 CpG sites identified in the literature (cg00300879, cg26764761, cg10864200, cg15414745, cg07080864, cg15079934, cg24049493).

In Model 1, we adjusted for age, sex, and center (Arizona, Oklahoma, and North Dakota/ South Dakota) and immune cell subtypes. In Model 2, we included all variables from Model 1, alcohol (never, ever, current), smoking (never, ever, current), BMI, and LDL-cholesterol. To examine the associations between circulating leukocyte subtypes and risk of liver cancer, we ran Cox proportional hazards models to determine HRs for each immune cell subtype. The HRs are interpreted as 1 SD increase in proportion for each immune cell subtype. All analyses were performed with R software (version 4.2.1).

## Results

Age, sex and center had similar distributions among liver cancer cases and non-cases (Table 1). The mean follow-up duration (SD) for non-cases was 25.1 (5.6) years and for cases, 11.0 (8.8) years. Cases had a higher prevalence of diabetes (47.6%) compared to non-cases (41.6%), although the difference was not significant. Similarly, there was no statistically significant difference between cases and non-cases for previously determined risk factors for liver cancer including alcohol use, smoking status, and BMI. LDL-cholesterol, on the other hand, was significantly higher in non-cases (120.5 mg/dL) compared to cases (102.8 mg/dL, *p* = 0.02).

**Table 1:** Participant baseline characteristics by liver cancer status, in the Strong Heart Study

Characteristic	Liver Cancer Status		*p*-value
	Cases	Non-cases	
	*n* = 21	*n* = 2,303	
Age, years, Mean (SD)	56.61 (8.72)	56.17 (8.09)	0.80
Sex, Male, No. (%)	8 (38.1%)	956 (41.5%)	0.75
Center, No. (%)			0.99
South Dakota	9 (42.9%)	1023 (44.4%)	
Oklahoma	9 (42.9%)	972 (42.2%)	
Arizona	3 (14.3%)	308 (13.4%)	
Diabetes, Yes, No. (%)	10 (47.6%)	957 (41.6%)	0.57
Smoking, No. (%)			0.38
Current	6 (28.6%)	887 (38.5%)	
Ever	7 (33.3%)	740 (32.1%)	
Never	8 (38.1%)	676 (29.4%)	
Alcohol, No. (%)			0.56
Current	10 (47.6%)	991 (43.0%)	
Ever	7 (33.3%)	972 (42.2%)	
Never	4 (19.0%)	335 (14.5%)	
Missing	0 (0%)	5 (0.2%)	
BMI, kg/m^2^, Mean (SD)	31.04 (6.90)	30.30 (6.08)	0.58
HDL cholesterol, mg/dL, Mean (SD)	50.62 (17.5)	46.33 (13.9)	0.16
LDL-cholesterol, mg/dL, Mean (SD)	102.8 (27.8)	120.5 (33.2)	0.02
Fasting glucose, mg/dL, Mean (SD)	141.7 (63.7)	138.9 (68.3)	0.85
Insulin, mIU/L, Mean (SD)	24.0 (14.2)	19.9 (21.9)	0.40
Total triglycerides, mg/dL, Mean (SD)	128.6 (61.7)	144.7 (107)	0.49

### Epigenome-wide analysis

**Fig. 1:**
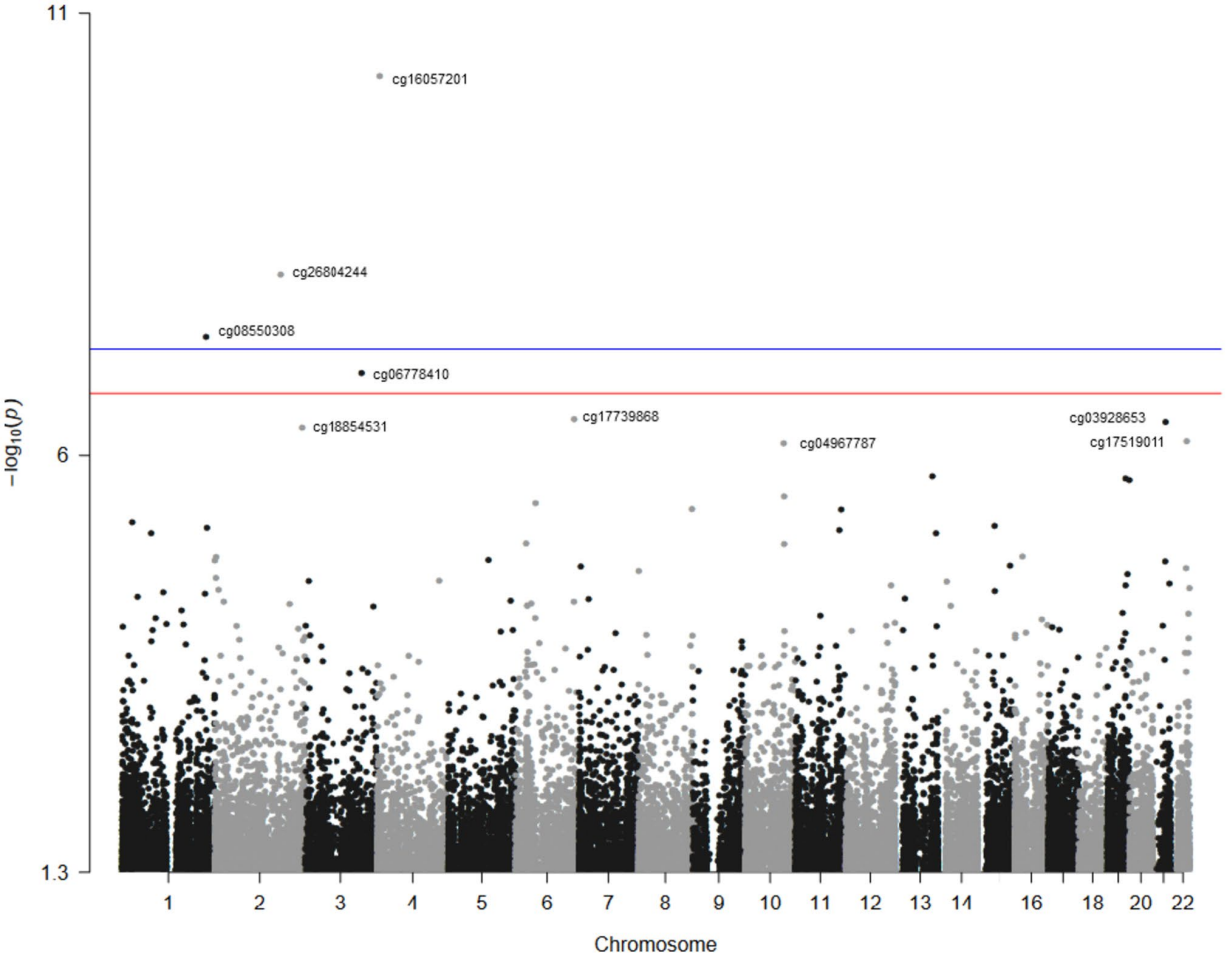
Manhattan plot of the Epigenome-Wide Association Study for liver cancer. The Manhattan plot of the Epigenome-Wide Association Study (EWAS) model. The *X* axis represents the chromosomal position, and the *Y* axis represents the significance on the – log 10 scale. The *p*-value is (1 × 10^–7^). The CpG sites further analyzed in this study are labelled

Figure 1 presents the EWAS Manhattan plot. A total of 9 CpG sites were significantly differentially methylated by liver cancer status (see beta value distributions in Supplemental Fig. 1 and Supplemental Table 1). One DMP (cg16057201) was hypermethylated in cases compared with non-cases and the other eight DMPs (cg26804244, cg08550308, cg06778410, cg17739868, cg03928653, cg18854531, cg17519011 and cg04967787) were hypomethylated in cases compared to non-cases (Table 2). The HR (95% CI) for a SD increase in methylation (Beta) for cg16057201 was 1.25 (1.14, 1.37) in model 2 adjusted for age, sex, study center site, alcohol use, smoking, BMI, LDL and immune cells. The corresponding HRs (95% CI) were 0.58 (0.44, 0.75) for cg04967787, 0.62 (0.47, 0.83) for cg06778410, 0.70 (0.59, 0.82) for cg26804244, 0.70 (0.56, 0.86) for cg17519011, 0.71 (0.60, 0.85) for cg18854531,0.75 (0.67, 0.84) for cg17739868, 0.75 (0.65, 0.87) for cg03928653, and 0.77 (0.67, 0.88) for cg08550308.

**Table 2:** Hazard ratios (HRs) and 95% Confidence Intervals (95%CI) for liver cancer by blood DNA methylation markers in the Strong Heart Study

CpG site	Model 1		Model 2		Associated genes	Gene functions
	HR (95% CI)	*p*-value*	HR (95% CI)	*p*-value		
cg16057201	1.25 (1.15, 1.36)	7.01E−08	1.25 (1.14, 1.37)	1.94E−06	MRFAP1	Cell growth
cg26804244	0.68 (0.58, 0.80)	3.69E−06	0.70 (0.59, 0.82)	2.57E−05		
cg08550308	0.77 (0.68, 0.87)	1.90E−05	0.77 (0.67, 0.88)	1.01E−04		
cg06778410	0.62 (0.47, 0.81)	6.08E−04	0.62 (0.47, 0.83)	1.04E−03		
cg17739868	0.74 (0.67, 0.82)	2.87E−08	0.75 (0.67, 0.84)	8.17E−07		
cg03928653	0.73 (0.63, 0.84)	1.91E−05	0.75 (0.65, 0.87)	8.75E−05		
cg18854531	0.71 (0.60, 0.83)	4.36E−05	0.71 (0.60, 0.85)	1.28E−04		
cg17519011	0.71 (0.58, 0.87)	1.11E−03	0.70 (0.56, 0.86)	8.06E−04	A4GALT	Globoside synthesis
cg04967787	0.53 (0.41, 0.70)	4.62E−06	0.58 (0.44, 0.75)	4.79E−05	PPRC1	Mitochondrial biogenesis

The HR is reported per SD of Beta for differential methylation markers

Model 1: age, sex, center, and immune cell proportions

Model 2: Model 1 + alcohol (never, ever, current), smoking (never, ever, current), BMI and LDL-cholesterol

### Blood cell types and liver cancer

Liver cancer cases had higher proportions of CD8+T cells, natural killer cells and monocytes than non-cases (Supplemental Fig. 2 and Supplemental Table 2). Higher proportion of CD8+T cells, natural killer cells, and B-lymphocytes at baseline were associated with increased liver cancer risk, however, none remain statistically significant after adjusting for other variables. (Table 3).

**Table 3:** HRs and 95% CI for immune cell proportions and liver cancer, in the Strong Heart Study

Immune cell subtype	Model 1		Model 2		Model 3	
	HR (95% CI)	*p*-value	HR (95% CI)	*p*-value	HR (95% CI)	*p*-value
CD8+	1.44 (0.99, 2.10)	0.06	1.46 (1.02, 2.10)	0.04	1.14 (0.70, 1.85)	0.61
CD4+	0.92 (0.59, 1.44)	0.72	0.96 (0.62, 1.51)	0.87	0.82 (0.50, 1.36)	0.45
Natural killer	1.55 (1.07, 2.26)	0.02	1.54 (1.05, 2.26)	0.03	1.41 (0.85, 2.33)	0.18
B-lymphocytes	1.36 (0.93, 2.00)	0.12	1.35 (0.93, 1.96)	0.12	1.37 (0.89, 2.12)	0.16
Monocytes	1.09 (0.71, 1.65)	0.7	1.03 (0.69, 1.56)	0.87	0.88 (0.55, 1.42)	0.61
Granulocytes	0.69 (0.44, 1.07)	0.09	0.67 (0.43, 1.04)	0.08	0.69 (0.41, 1.01)	0.1

The HR is reported per SD of proportion for each cell counts

Model 1: age, sex and center

Model 2: model 1 + alcohol (ever/current vs never), smoking (ever/current vs never), BMI and LDL

### Targeted models with DMPs from previous studies

We assessed 7 candidate CpG sites from 2 prior studies, assessing blood DNA methylation and hepatocellular carcinoma [16, 17]. The HRs per SD increase in methylation and 95% CIs for Model 2, was 1.93 (1.1, 3.39) (*p* = 0.02) for cg15079934 (Table 4).

**Table 4:** Hazard ratios and 95% confidence intervals for candidate CpGs from literature and liver cancer, in the Strong Heart Study

CpG site	Model 1		Model 2	
	HR (95% CI)	*p*-value*	HR (95% CI)	*p*-value
cg00300879	1.12 (0.72, 1.76)	0.37	1.12 (0.70, 1.77)	0.64
cg26764761	1.07 (0.68, 1.67)	0.79	1.03 (0.67, 1.61)	0.87
cg10864200	1.50 (0.89, 2.52)	0.06	1.51 (0.90, 2.52)	0.12
cg15414745	1.15 (0.72, 1.85)	0.71	1.13 (0.69, 1.84)	0.66
cg07080864	0.89 (0.57, 1.38)	0.73	0.87 (0.55, 1.36)	0.54
cg15079934	1.95 (1.11, 3.43)	0.03	1.93 (1.1, 3.39)	0.02
cg24049493	0.94 (0.59, 1.49)	0.73	0.90 (0.56, 1.45)	0.62

The HR is reported per SD of Beta for candidate CpGs

Model 1: age, sex, center, and immune cell proportions

Model 2: Model 1 + alcohol (never, ever, current), smoking (never, ever, current), BMI and LDL-cholesterol

## Conclusion and discussion

Our study found alterations in DNA methylation of selected CpG sites at baseline associated with liver cancer mortality including hypermethylation in cg16057201 (annotated to *MRFAP1*) and hypomethylation in 8 CpG sites. Our study found alterations in blood DNA methylation profiles are associated with liver cancer death, suggesting that DNA methylation data may potentially be useful as a biomarker to identify high-risk individuals for effective primary prevention, and for risk-based screening options.

We found no significant difference between cases and non-cases for most established risk factors for liver cancer such as smoking, diabetes, and BMI. However, we found participants with liver cancer had a statistically significant lower mean LDL-cholesterol level (102.8 mg/dL) compared to participants without liver cancer (120.5 mg/dL). A cohort study following Japanese adults [27] also found an association between low LDL-cholesterol level and liver cancer mortality and extrapolated that low LDL-cholesterol levels could be a predictive marker for liver cancer death [27]. Previous studies have reported that people with cirrhosis have low serum LDL-cholesterol levels [28–30]. Lower LDL-cholesterol at baseline in participants who later on died of liver cancer cases might reflect the underlying liver cirrhosis condition.

Despite the significant associations with risk of liver cancer, only 3 of the 9 CpG sites are located in protein coding regions of genes; cg16057201 is located on chromosome 4 in a CpG island region within *MRFAP1*. *MRFAP1* encodes a protein that is involved in maintaining normal histone modification levels by downregulating recruitment of the NuA4 complex to chromatin [31]. There is decreased protein expression of MRFAP1 in gastric cancer cells as compared to non-cancerous cells. When MRFAP1 knockout gastric cancer cells were treated with MLN4924, a neddylation inhibitor under investigation as a cancer treatment, increased cytotoxicity was observed, suggesting that MRFAP1 may be protective against MLN4924 [31]. While *MRFAP1* is post-translationally regulated, hypermethylation at this CpG site could decrease the expression of this protein, especially as this CpG site is located in the promoter region, potentially impacting the regulation of cell growth. cg17519011 is located on chromosome 22 in a south of the shore region of *A4GALT*. *A4GALT* encodes an enzyme, α1-4 galactosyltransferase that helps to synthesize globoside glycosphingolipids (GSLs) which are important for cellular recognition in cell–cell adhesion [25]. *A4GALT* has elevated expression in epithelial ovarian cancer cells which is associated with positive patient prognosis. However, overexpression of GSLs, more specifically globotriaoslyceramide *Gb3*, which is catalyzed by *A4GALT*, is associated with tumorigenesis in breast and gastric cancer. cg049677787 is located on chromosome 10 in *PPRC1* in the open sea region. The protein encoded by *PPRC1* acts as a coactivator in transcriptional activation of nuclear genes related to mitochondrial biogenesis and energy metabolic processes. The biological implications of hypomethylation in the other 6 CpG sites are unclear. Further research could evaluate the effect of methylation in gene coding regions on liver cancer development.

High proportions of CD8+T and NK cells in circulating leukocyte profiles were associated with liver cancer risk. Cytotoxic CD8+T cells play an important role in the adaptive immune system but can undergo T cell exhaustion characterized by loss of effector function and overexpression of inhibitory receptors within cancer conditions [30]. In patients with hepatocellular carcinoma (HCC), the most common form of liver cancer, exhausted CD8+T cells were present in tumor tissue and peripheral blood with greater frequency than patients without HCC [30]. Patients with non-alcoholic steatohepatitis (NASH) or HCC showed increased amounts of CD8+ and NK T cells compared with controls, implying an important role in steatosis and cancer development when interacting with hepatocytes [31]. In the Strong Heart Study, metabolic syndrome, which affects more than 50% of the population, is a risk factor for fatty liver disease [32].

Among the 7 CpG sites found in literature, we found hypermethylation in cg15079934 was associated with liver cancer which was consistent with previous study [17]. There were discrepancies between the literature and our study in the direction of methylation associated with HCC which could be attributed to many factors including differences in population location, HBV status, and different leukocyte proportions. However, hypermethylation at cg15079934 (annotated to *LSP1*) was associated with HCC among patients with liver cirrhosis [17], suggesting that this DNA methylation marker might be potentially used as marker of HCC. Low expression of LSP1 was correlated with poor clinicopathological features such as large tumor size, and advanced tumor-node-metastasis (TNM) stage in HCC tumors [33]. Another explanation for the discrepancy between our findings and prior studies is the model adjustments and computational methods used in our and prior studies. In the prior two studies used Wilcoxon signed-rank, while we use linear regression models in the EWAS analysis.

There are several limitations in our study. First, the major limitation of our study is the lack of data on HBV and HCV infection in participants in the Strong Heart Study. As HBV and HCV are common causes of liver cancer, knowing the infection status of participants could inform further research. Second, more granular measures of alcohol consumption, as an established risk factor, were not available for the majority of the study population, which may contribute to residual confounding. Due to the exclusion criteria, it is possible that the study population contains a lower proportion of individuals who smoke or drink alcohol, as those behavioral risk factors are associated with both liver cancer and cardiovascular disease. Other limitations of this study include the limited number of liver cancer cases and lack of a validation cohort. In addition, the HRs were standardized by a one standard deviation (SD) increase in methylation (beta value) to control for the small sample size and outliers, resulting in difficulty interpreting the results.

However, to our best knowledge, this is the first and the largest cohort study focusing on an American Indian population. Our study is particularly important because of an increasing trend of liver cancer among American Indians and Alaska Natives [34]. We provide evidence that aberrant methylation in leukocyte DNA is associated with increased risk of liver cancer, suggesting the potential use of DNA methylation as a marker for identifying high-risk individuals. Moreover, the focus on American Indian populations helps to promote inclusion for peoples historically excluded from scientific and medical research. Centering American Indian populations supports health equity, as we aim for our research to help in mitigation and prevention of liver cancer. The main study population in the Strong Heart Study included 4549 men and women. Our study only used information from 2350 participants and excluded participants for several reason including without sufficient urine sample for metal determination and with cardiovascular disease [22]. As the exclusion criteria were not related to cancer outcome, it is unlikely that the findings from our current study can be explained by selection bias.

In summary, our EWAS of liver cancer suggests that DNA methylation may be associated with liver cancer and circulating leukocyte profiles may be altered before liver cancer. These conclusions should be validated by a larger study with a larger sample size of liver cancer cases.

### Supplementary Information

Below is the link to the electronic supplementary material.

Supplementary file1 (DOCX 124 KB)

## Data Availability

The data underlying this article cannot be shared publicly in an unrestricted manner due to limitations in the consent forms and in the agreements between the Strong Heart Study tribal communities and the Strong Heart Study investigators. The data can be shared to external investigators following the procedures established by the Strong Heart Study, available at https://strongheartstudy.org/. All analyses were conducted in R version 3.6.2 and all packages used are freely available in the CRAN repository.

## Abbreviations

BMI: Body mass index
CpG: Cytosine guanine dinucleotide
EWAS: Epigenome-wide association study
HRs: Hazard ratios
LDL: Low-density lipoprotein
NK: Natural killer
SD: Standard deviation
SHS: Strong Heart Study
DMP: Differentially methylated position
