# Supplementary material for: Blood DNA methylation and liver cancer in American Indians: evidence from the Strong Heart Study
Source: Cancer Causes Control. 2023 Nov 27;35(4):661–9. doi: 10.1007/s10552-023-01822-8 (PMC10960679; doi:10.1007/s10552-023-01822-8)
Supplement: Supplementary file 1 — Supplementary file1 (DOCX 124 KB) [file 10552_2023_1822_MOESM1_ESM.docx]

**Supplemental Figures**

**Supplemental Figure 1:** The Distributions of 9 CpG sites by Liver Cancer Status, in the Strong Heart Study


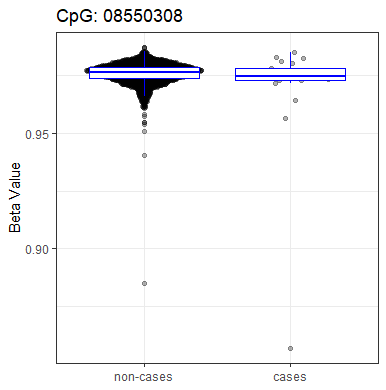

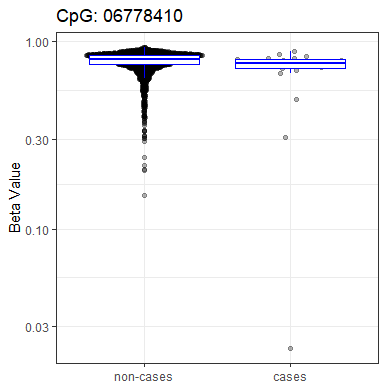


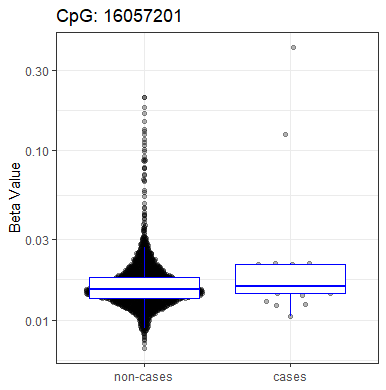

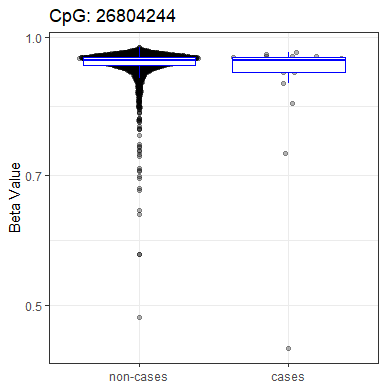


**
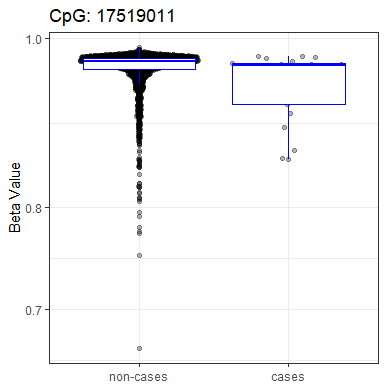
**


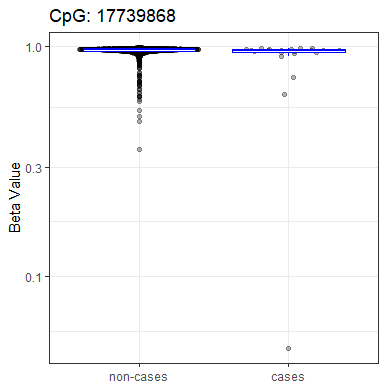

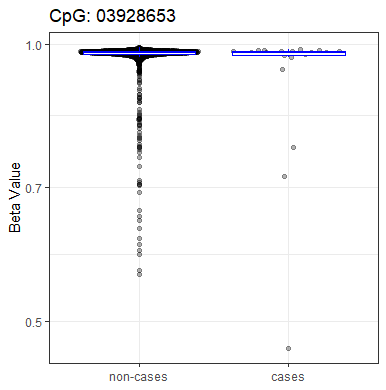


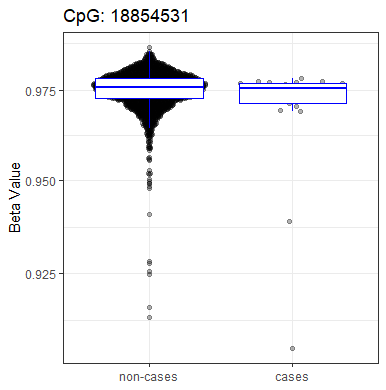


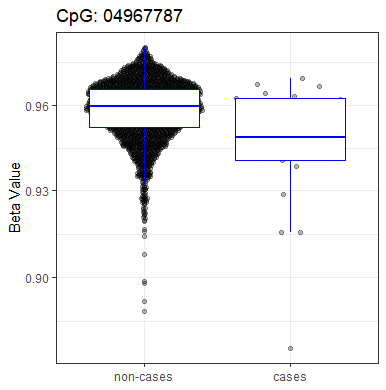


**Supplemental Figure 1 Legend:** Box plots compare the spread of percent methylation of EWAS-identified significant CpG sites by liver cancer status.

**Supplemental Figure 2**: The Distributions of Immune Cell Proportions by Liver Cancer Status, in the Strong Heart Study


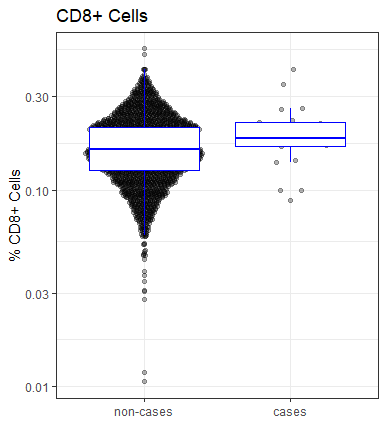

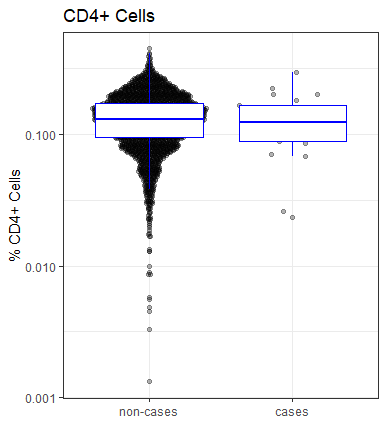


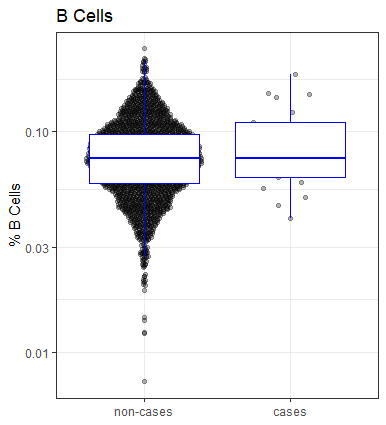

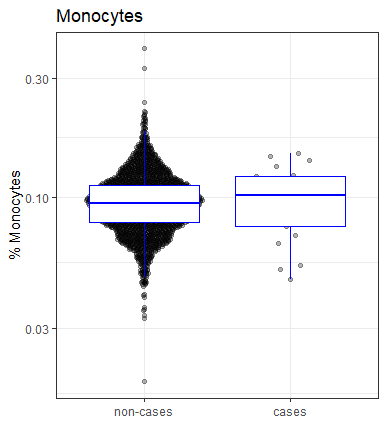


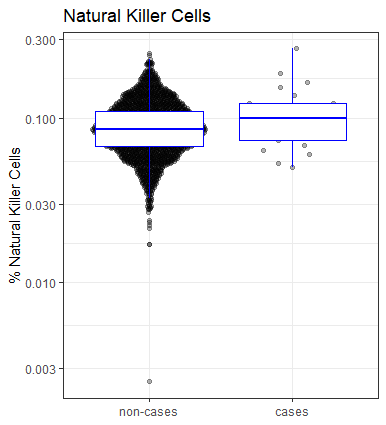

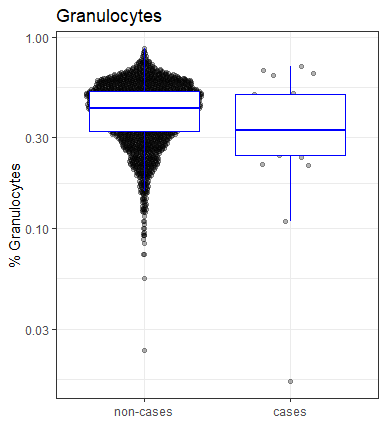


**Supplemental Figure 2 Legend:** Box plots compare the proportion of immune cell subtype by liver cancer status. The proportions were ascertained by the Houseman computational method.

**Supplemental Figure 3:** Spearman Correlation of Immune Cell Subtypes**
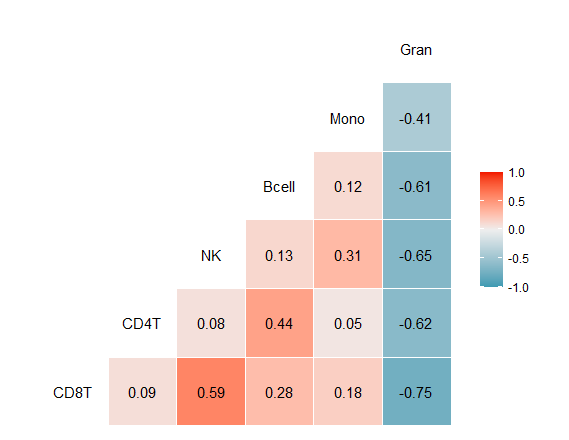
**

**Supplemental Figure 3 Legend:** The spearman correlation assesses the strength and direction of the monotonic relationships between all combinations of immune cell type included in this study.

**Supplemental Table 1**: The Distribution of log Fold Chage of 9 CpG Site by Liver Cancer Status

| CpG Site | logFC | P-value | Chromosome | Gene |
| --- | --- | --- | --- | --- |
| cg16057201 | 1.13 | 5.29E-11 | 4 | MRFAP1 |
| cg26804244 | -0.70 | 9.14E-09 | 2 |  |
| cg08550308 | -0.44 | 4.61E-08 | 1 |  |
| cg06778410 | -0.86 | 1.18E-07 | 3 |  |
| cg17739868 | -1.12 | 3.90E-07 | 6 |  |
| cg03928653 | -0.97 | 4.20E-07 | 21 |  |
| cg18854531 | -0.39 | 4.86E-07 | 2 |  |
| cg17519011 | -1.08 | 6.92E-07 | 22 | A4GALT |
| cg04967787 | -0.49 | 7.32E-07 | 10 | PPRC1 |

**Supplemental Table 2**: Immune Cell Subtype Counts by Liver Cancer Status

|  | **Liver Cancer Status** | |  |
| --- | --- | --- | --- |
|  | **Cases** | **Non-Cases** |  |
| **Immune Cell Subtype, Median (IQR)** | **N = 21** | **N = 2303** | **P-VALUE** |
| CD8+ | 0.185 (0.055) | 0.162 (0.084) | 0.06 |
| CD4+ | 0.123 (0.079) | 0.131 (0.079) | 0.70 |
| Natural Killer | 0.099 (0.050) | 0.086 (0.043) | 0.03 |
| B-lymphocytes | 0.076 (0.048) | 0.076 (0.039) | 0.11 |
| Monocytes | 0.102 (0.045) | 0.094 (0.032) | 0.80 |
| Granulocytes | 0.326 (0.259) | 0.424 (0.200) | 0.10 |

P values were calculated from ANOVA test
